# Localised breast cancer: neoadjuvant chemotherapy impact evaluation on the pathological complete response (PCR) in a lower middle-income country

**DOI:** 10.3332/ecancer.2023.1648

**Published:** 2023-12-15

**Authors:** Ganiou Adjadé, Hassan Abdelilah Tafenzi, Houda Jouihri, Nadin Shawar Al Tamimi, Yousra Bennouna, Gustave Négamiyimana, Kalil Cisse, Ismail Essadi, Mohammed El Fadli, Rhizlane Belbaraka

**Affiliations:** 1 Department of Medical Oncology, Mohammed VI University Hospital, 2360 Marrakech-principal, Morocco; 2 Biosciences and Laboratory, Faculty of Medicine and Pharmacy, Cady Ayyad University, 7010, Sidi Abbad, Marrakech 40000, Morocco; 3 Department of Medical Oncology, Avicenna Military Hospital, Marrakech 40000, Morocco; a https://orcid.org/0000-0003-0768-7493

**Keywords:** chemotherapy, neoadjuvant, breast cancer, localised, PCR, Morocco

## Abstract

**Introduction:**

Neoadjuvant chemotherapy followed by surgery remains the current recommended therapeutic strategy for the management of locally advanced breast cancer. The standard chemotherapy protocol is sequential with anthracycline followed by taxanes. However public hospitals in Morocco are confronted with a shortage in healthcare products. We, therefore, evaluated the efficacy of the different protocols proposed to patients, by evaluating the clinical response after chemotherapy especially through the pathological complete response (PCR) after surgery.

**Methods:**

We focused on the database of the medical oncology department of the only public hospital covering middle and southern Morocco. We included patients diagnosed between 2018 and 2020. Two groups of patients distinguished in group A (GA) received the standard therapy, and group B (GB) received a non-standard protocol. The therapeutic response was assessed clinically before surgery and then by pathological examination of the surgical specimens. The Sataloff classification defined the histological response for both tumour and lymph nodes. We included both groups in one variable to determine their impact on outcomes. A logistic regression-based analysis was adopted to define variables related to the PCR.

**Results:**

Over the study period, 120 patients met our inclusion criteria. They were distinguished at 72% in GA and 28% in GB. 60.8% over 120 got a partial response, whereas, only 33.3% achieved a complete response. The general PCR rate was 28% with 14.3% in GB and 85.7% in GA. The tumour PCR was obtained in 40 (33.3%) over 120 patients and 51.7% of lymph node PCR. The multivariate logistic regression analysis results show no relative factors associated with general PCR achievement.

**Conclusion:**

These first interesting results from an institutional database inform us about our clinical practice and require additional research using prospective randomised controlled studies.

## Highlights

- BC is the most common cancer in women but public hospitals in Morocco are confronted with a shortage in the supply of healthcare products.
- We, therefore, evaluated the efficacy of the different protocols proposed to patients, through the evaluation of the clinical response after chemotherapy and especially through the PCR after surgery.
- Two groups of patients distinguished in GA received the standard therapy and GB received a non-standard protocol according to the availability of chemotherapy molecules
- The general PCR rate was 28% with 14.3% in GB and 85.7% in GA
- Alternative chemotherapy protocols to the therapeutic standard are less effective in terms of PCR and accessibility to the best treatment must be a priority.

## Introduction

Over the past decades, neoadjuvant therapy has become a preferred option in locally advanced, [1] inflammatory, node-positive, triple-negative (TN), or human epidermal growth factor receptor 2 (HER2) – overexpressing, non-metastatic breast cancers (BCs) [2–4]. It has the advantage of being able to induce a reduction in tumour size [5], and provides valuable prognostic information, as patients achieving a pathological complete response (PCR) have significantly longer disease-free survival (DFS) and overall survival (OS) [6, 7]. In addition, postoperative therapy decisions are affected by pathologic response to neoadjuvant therapy.

This sequence followed by surgery remains the current recommended therapeutic strategy for the management of locally advanced BC. The standard chemotherapy protocol is a sequential treatment with an anthracycline/epirubicin + cyclophosphamide (AC or EC) followed by taxanes (T), with or without anti-human epidermal growth factor receptor 2 (anti-HER2) treatment depending on the expression of the HER2 protein on immunohistochemistry (IHC) [5, 8].

Morocco’s health system still faces financial and organisational difficulties. For years, the Kingdom has been making efforts to finance the health sector, notably by strengthening the policy of basic medical coverage. This policy’s social impact has led to the introduction of the Medical Assistance Scheme (Régime de l’Assistance Médicale, or RAMED), a national social cover for the benefit of the most disadvantaged. This insurance allows our vulnerable patients to benefit free of charge from all medically required services available in the country's public health structures. However, most patients still consult at the late stages of the disease [9, 10].

Public hospitals, faced with increasing demand, are confronted with a shortage in the supply of healthcare products. The patients followed in our medical oncology department are vulnerable. They are unable to afford optimal care without RAMED. Indeed, the minimum wage in Morocco is 265 euros, whereas the cost of chemotherapy treatment in our context is 290 euros for a course of chemotherapy with anthracycline, 345 euros for paclitaxel, 455 euros for docetaxel, and 1,500 euros for a course of chemotherapy with taxane and anti-HER2 (trastuzumab). Moreover, as multidisciplinary consultation meetings are not yet compulsory, most patients are operated on immediately.

Localised BC treatment, in a neo-adjuvant approach, appears to be the ideal indicator to evaluate the effectiveness of our treatment according to the molecules available and accessible in our health structure. We, therefore, wanted to evaluate the efficacy of the different protocols proposed to patients through the evaluation of the clinical response after chemotherapy and especially through the PCR after surgery.

## Methods

### Data source

We focused on the database of the medical oncology department of the Mohammed VI University Hospital of Marrakech in Morocco. This department is the only public clinical oncology covering middle and southern Morocco and offers the international standard accepted clinical care.

### Study design and population

This is a descriptive, analytical, retrospective study over 33 months between January 2018 and September 2020, concerning patients above 18 years diagnosed with BC as a primary malignancy tumour, and going for neoadjuvant treatments, with different clinical stage T (1a–4d), N (1–3), and different HER2 status.

Eligible patients were those who received neoadjuvant chemotherapy and then underwent surgery either mastectomy or tumorectomy or lumpectomy. Moreover, patients were excluded if: 1) they received neo-adjuvant endocrine therapy alone, or 2) they underwent excisional biopsy of the tumour; or 3) patients without precise pathological data or surgical information, and patients lost to follow-up or died during treatments without any specific cause.

Neoadjuvant chemotherapy was administered according to the standard protocol [11], and the availability of chemotherapy drugs.

Two groups of patients were distinguished:

- Group A (GA) received the standard therapy of AC (in AC60, EC100, or FEC100 protocols) followed by taxanes +/- anti-HER2 treatment.
- Group B (GB) of patients received a non-standard protocol according to the availability of chemotherapy molecules. We grouped patients who received the Taxane-anthracycline sequence (TAS), a monotherapy consisting of anthracyclines or taxane +/- anti-HER2 treatment (Mono) or a mixed protocol (Mixed) consisting of a sequence of anthracycline followed by Taxanes and vice versa according to the availability of the molecules.

### Immunohistochemical profile

We distinguished four immunohistochemical subtypes: Luminal A, Luminal B, HER2, and TN profiles.

Depending on the marking of the different receivers, we have defined these different profiles as follows

- Luminal A: expression of oestrogen (ER+) and/or progesterone (RP+) receptors, absence of expression of the HER2 protein (HER2-) with a Ki67 <14%
- Luminal B: RE+ and/or RP+, with a Ki67 >14% regardless of the expression of the HER2 protein
- HER2 positive: RE- and RP- with high expression of the HER2 protein (HER2 3+)
- TN: absence of expression of the three proteins (RE-, RP- and HER2-) [12].

### Pathologic response

The therapeutic response was assessed clinically before surgery and then by pathological examination of the surgical specimens. The Sataloff classification [13] was used to define the histological response for both tumour and lymph nodes.

The PCR was defined as the absence of invasive tumour residue (ypT0) in the breast and invaded nodes (ypN0). Patients with residual ductal carcinoma *in situ* with no evidence of residual invasive disease were included in this category [14].

The PCR was the primary endpoint and was categorised into the proportion of patients who achieved a PCR on the tumour PCR (TPCR) or PCR lymph nodes (NPCR). In addition, the variable PCR was added as a factor, and defined as a union between TPCR and NPCR, given the positivity of each one, implicating the positivity of PCR.

### Covariates

Demographic, clinic, pathologic, and therapeutics covariates were selected for the analysis to assess factors associated with either TPCR, NPCR, or PCR. This includes sex, age at diagnosis, laterality, histology type, percentage expression of hormonal receptors (oestrogen and progesterone), HER2 status, immunohistochemical profile (positive HER2, Luminal A, Luminal B, or TN) BC, treatment groups (A, B); Therapeutic strategies (Standard, TAS, Mono, Mixed); the time interval between last chemotherapy cure and surgery; response evaluation [complete, partial, no clinical response (NCR)]; surgery type (tumorectomy or lumpectomy, mastectomy by Patey); Scarff-Bloom-Richardson (SBR) grading, vascular emboli (positive, negative); both clinical and pathological T stage, N stage; Sataloff classification. The presented data concern only the neoadjuvant field of the first-line chemotherapy course.

### Statistical analyses

Since all patient characteristics were categorical variables, they were described using frequency statistics, except the continuous covariate age at diagnosis which was expressed using median and interquartile ranges. We reported chemotherapy treatments separately using family drug anthracyclines and/or taxanes. Moreover, we reported separately HER2 covariates to stratify and compare the associating and relative impact of therapeutic regimes on the frequency of PCR, TPCR, and NPCR. Factors related to the three outcomes were assessed using univariate and multivariate logistic regression models that include PCR, TPCR, and NPCR as three dependent variables separately, and patients’ characteristics as independent variables. We included both groups GA and GB in one variable to determine their impact on outcomes. Individuals with more than two covariates missing were excluded from the analyses. All statistical analyses were done using R Software Version 3.2. A *p*-value less than 0.05 was considered statistically significant.

## Results

### Patients’ characteristics

In this study, we included 120 patients (11.6%) out of 1,034 patients diagnosed between 2018 and 2020 with BC as a primary malignancy tumour. They were treated with neoadjuvant chemotherapy followed by surgery (Table 1). The median age at diagnosis was 49 years. 98.3% (*n* = 118) of them were female, with the left breast as the affected site (*n* = 69), diagnosed with ductal carcinoma at 92.5% (*n* = 111), and luminal B (*n* = 69) as the most frequent immunohistochemical profile at 57.5%.

At diagnosis, 47.5% (*n* = 57) of patients were classified T_4b_ following the clinical tumour staging characterised by the presence of a macroscopic ulceration, orange peel, and in some cases, 16.7% (*n* = 20) presented with an inflammatory sign, associated with the presence of a mobile lymph node metastasis in I/II level in 41.7% (*n* = 50) of patients, without any indication of distance metastatic organs.

Regarding treatment characteristics, 71.7% (*n* = 86) received standard treatment, followed by breast removal including a variable number of axillary lymph nodes or Patey 82.5% (*n* = 99). 73 patients (*n* = 60.8%) over 120 got a partial response (PaR), whereas, only 33.3% (*n* = 40) achieved a complete response. The median time interval between the last chemotherapy course and surgery was 8 weeks. The majority of tumours were intermediate-grade SBR II (*n* = 72; 60%). Lymph vascular invasion (LVI) was reported in 31.7% (*n* = 38) of the cases.

### Concerning TPCR

The TPCR was obtained in a total of 40 (33.3%) over 120 patients. Following surgery, 38.3% (*n* = 46) of patients were classified at T_2_, and only 26.7% (*n* = 32) were found without tumour residue. Concerning the Sataloff classification of the tumour, 44 patients (36.7%) were classified as TB (grade B: more than 50% therapeutic effect but less than total or near-total effect) followed by TA (total or near-total therapeutic effect,) in 30% (*n* = 36) of cases. Meanwhile, 10% (*n* = 12), were classified TD (no therapeutic effect) and did not benefit from the chemotherapy. Based on the results of univariate logistic regression analysis, patients who received mixed protocol between two different chemotherapy agents were associated with the outcome, in addition to oestrogen and progesterone expression, HER2 status, immunohistochemical profile, treatments group, pathological N category, and Sataloff. Finally, Sataloff remains the only independent variable correlated with TPCR (Table 2).

### Concerning lymph node PCR

The lymph node PCR was obtained in a total of 62 (51.7%) over 120 patients, which was much higher than the results of TPCR. 45.8% (*n* = 55) were found without lymph node metastasis which is an indication of achieving the therapeutic effect, thus they were classed NA according to lymph node Sataloff classification. In the univariate adjusted analysis, oestrogen-expressing receptors, HER2 status, and Sataloff were factors associated with NPCR obtention. However, in the multivariate-adjusted analysis, ER percentage expression and Sataloff were related to greater odds of achieving NPCR (Table 3).

### Concerning the general PCR

The variable PCR was created to define a general result that includes the combination of TPCR, and NPCR, given the correlation of each TPCR, or NPCR with the likelihood of PCR. The general PCR rate was 28% with 14.3% in GB and 85.7% in GA. The multivariate logistic regression analysis results show no relative factors associated with general PCR achievement (Table 4).

## Discussion

Through this retrospective study, we report the experience of the largest hospital center in central Morocco. This experience also aims to enlighten oncologists in developing countries on the possible therapeutic options in the context of difficult access to chemotherapy molecules.

In Africa and Morocco, in particular, the diagnosis and treatment of cancer are very expensive for patients, especially given the social standard of living. This social reality explains the delay in the first consultations, in the diagnosis, and also the difficulties of treatment.

Over the past decades, neoadjuvant therapy has become preferred in locally advanced, inflammatory, non-metastatic BCs, especially in node-positive, TN, or HER2-overexpressing tumours [8, 15].

During our study period, 120 patients met these criteria. They presented a locally advanced tumour in 67.6% of cases, and an inflammatory in 16.7% of cases with 55.9% lymph node invasion.

Of 1,034 patients diagnosed between 2018 and 2020 for BC, only 120 of them have been eligible for this study. The main objective element was the evaluation and prognostic comparison value of different treatment protocols to obtain the PCR in general, TPCR, and NPCR in specific. For that, and through univariate and multivariate logistic regression analysis, we found that ER percentage expression and Sataloff classification were related to greater odds of achieving NPCR.

The results from large randomised clinical trials have shown that neo-adjuvant and adjuvant chemotherapy is associated with similar long-term outcomes [5, 16, 17]. Preoperative chemotherapy has the advantage of being able to induce a reduction in tumour size [5, 16]. Furthermore, the antitumor activity of neoadjuvant chemotherapy can provide valuable prognostic information, as patients achieving a PCR have significantly longer DFS and OS, especially for TN or HER2+ BC [18, 19].

The current standard in a neoadjuvant situation is sequential treatment by adding taxanes to the anthracycline protocol combining doxorubicin with cyclophosphamide as in the B-27 trial [20] which demonstrated an improvement in the PCR rate of 27% against 13% in the B-18 trial [21] where patients have been treated only with an anthracycline [22].

We divided our patients into two groups according to the treatment received. 86 patients (71.7%) were able to benefit from the therapeutic standard in GA while 28.3% of GB were treated according to the availability of chemotherapy molecules. In GB, 8 patients (6.7%) received the reverse of the standard (TAS protocol), 12 patients (10%) were treated with a monotherapy made up only of anthracycline or taxane and 14 patients (11.7%) treatment seekers received a mixed protocol.

At the clinical evaluation after the courses of chemotherapy, a complete clinical response was observed in 33.3% of the cases, a PaR in 60.8% of the cases with a tumour reduction of 50% and a disappearance of the inflammatory signs. Unfortunately, four patients (3.3%) reported a lack of clinical response.

The overall PCR rate was 28% distributed as 24% in GA and 4% in GB (Mixed 1%; Mono 2%; STA 1%)

Sataloff's classification was an independent variable associated with both TPCR and NPCR. It allowed us to distinguish 33.3% TPCR and 51.7% NPCR. Moreover, and in association with the Sataloff T stage, ER was also associated with the obtention of NPCR. We excluded the pathologic T stage from subsequent analysis because it causes bias in the univariate and multivariate regression analysis. Moreover, we also decided to exclude the pathologic N stage from the final multivariate logistic regression analysis in the TPCR because it causes bias in the final model to select the variable related to it. Interestingly, and based on the results of multivariate logistic regression analysis, no independent variable has an impact on general PCR, this could be explained by the limited sample used.

The expression of hormone receptors and overexpression of HER2 appeared as factors associated with a better PCR, i.e., 13% in luminal B and 10% in HER2+. We did not obtain a PCR in the luminal A population; while only 5% of the TNs reported a PCR.

Our results, although encouraging, are far from those reported in the literature. This is particularly the case in the HER2 population with 21.5% in the NeoSphère trial [23], 27% in the NeoALTTO trial [24], 37% by An *et al* [25], and 45% in the CALBG 40601 trial [26].

The American College of Surgeons Oncology Group's Z1071 study [27] found that the PCR rate was lowest in luminal-type BCs (11.4%), with an intermediate value for the TN subtype (38.2%).

Clinical stage T and lymph node involvement are prognostic factors commonly used to determine the type of chemotherapy to be administered in a neoadjuvant setting, whether monotherapy or combination chemotherapy [25]. In our study, we did not take these criteria into account. Our patients received chemotherapy according to the availability of molecules. Moreover, response to chemotherapy is a biologically informative prognostic marker and might be a better stratification point to indicate which patients need more or less aggressive systemic treatment [25]. This is all the more indicative since we only obtained a 2% PCR rate in patients subjected to mono chemotherapy.

It should be noted that the PCR rate in our study is also influenced by the delay of surgery. This delay explains the absence of PCR in some patients who received the appropriate therapeutic protocol with an increase in the absolute risk of lymph node invasion of 5.3% in patients operated in a delay of more than 12 weeks as reported by Khader *et al* [28] This delay could be explained by the increasing number of patients in the surgery departments, the organisational difficulties in scheduling surgeries, and the difficulties for some patients in carrying out the pre-surgical assessment in time. Tjoe *et al* [29] also reported factors such as age, availability of health insurance and pre-surgery assessment such as magnetic resonance imaging.

In addition, postoperative therapy decisions are affected by pathologic response to neoadjuvant therapy. Thus, patients with residual invasive carcinoma after neoadjuvant chemotherapy will be offered capecitabine as an adjuvant in the TN population as highlighted in the CREATE-X trial [30], or trastuzumab emtansine (TDM1) in HER2+ patients as shown in phase 3 Katherine study [31].

This study has several important limitations. Important clinical data were missing such as certain immunohistochemical profiles and the clinical response after courses of chemotherapy. We did not provide information on the number of cures received and the chemotherapy protocols for the group of patients submitted to the mixed protocol. In addition, a correlation must be established between the clinical response and the PCR to inform patients about the therapeutic plan post-surgery. An effort must be made to comply with the therapeutic standard and the authorities at various levels are asked to make chemotherapy molecules available and in sufficient quantity. The therapeutic circuit of patients with BC and discussions in the multidisciplinary meeting must also be optimised to reduce the delay in surgical management.

## Conclusion

Our retrospective paper shows exactly the variables related to the obtention of PCR after neoadjuvant therapy with different treatment approaches, but it lacks specific prognostic evidence. In contrast to most of the prior randomised trials addressing the predictive and prognostic values of various therapy strategies, our study was based on real-life data outside of the clinical trial setting. Additional research using prospective randomised controlled studies is required to verify these findings.

## List of abbreviations

AC, Anthracycline + cyclophosphamide; BC, breast cancer; DFS, disease-free survival; EC, epirubicin + cyclophosphamide; ER, oestrogen receptor; GA, group A; GB, group B; HER2, human epidermal growth factor receptor 2; IHC, immunohistochemistry; LVI, lymphovascular invasion; NCR, no clinical response; NPCR, PCR in the lymph nodes; OS, overall survival; PCR, pathological complete response; PR, progesterone receptor; PaR, partial response; SBR, Scarff, Bloom, and Richardson, histo-prognostic grade; TAS, taxane-anthracycline sequence; TN, triple-negative; TPCR, tumour PCR.

## Conflicts of interest

The authors declare that they have no competing interests.

## Consent for publication

Not applicable.

## Funding

None.

## Ethical approval

The Marrakech Faculty of Medicine and Pharmacy's Ethical Review Committee gave its approval for data access. It was not necessary to obtain the patient’s informed permission. Before analysis, patient records were anonymised to ensure confidentiality. All methods were performed according to the relevant guidelines and regulations.

## Availability of data and materials

The datasets used and/or analysed during the current study are available from the corresponding author upon reasonable request.

## Author contributions

Substantial contributions to conception and design, acquisition of data or analysis and interpretation of data: Ganiou Adjadé, Hassan Abdelilah Tafenzi, Houda Jouihri, Ismail Essadi, Mohammed El Fadli and Rhizlane Belbaraka. Drafting the article and revising it critically for important intellectual content: Ganiou Adjadé, Hassan Abdelilah Tafenzi, Houda Jouihri, Ismail Essadi, Mohammed El Fadli and Rhizlane Belbaraka. Final approval of the version to be published: Ganiou Adjadé, Hassan Abdelilah Tafenzi, Houda Jouihri, Ismail Essadi, Mohammed El Fadli and Rhizlane Belbaraka. Agreement to be accountable for all aspects of the work in ensuring that questions related to the accuracy or integrity of any part of the work are appropriately investigated and resolved: Ganiou Adjadé, Hassan Abdelilah Tafenzi, Houda Jouihri, Ismail Essadi, Mohammed El Fadli and Rhizlane Belbaraka. Administrative support: Medical Oncology Department, Mohammed VI University Hospital, Marrakech, Morocco. Provision of study materials or patients: Rhizlane Belbaraka.

## Figures and Tables

**Table 1.** Descriptive characteristics of BC patients.

Characteristics	*N*	%
Sex		
Female	118	98.3
Male	1	0.8
Missing value	1	0.8
Age		
Median (min-max)	49 (27–80)	
Laterality		
Bilateral	2	1.7
Right	48	40.0
Left	69	57.5
Missing value	1	0.8
Histology		
Ductal	111	92.5
Lobular	7	5.8
Missing value	2	1.7
ER	85	70.8
Missing value	35	29.2
PR	92	76.7
Missing value	28	23.3
HER2		
HER2-	80	66.7
HER2+	38	31.7
Missing value	2	1.7
IHC		
HER2+	21	17.5
Luminal A	8	6.7
Luminal B	69	57.5
TN	20	16.7
Missing value	2	1.7
Group		
GA	86	71.7
GB	34	28.3
Protocol		
Mixed	14	11.7
Mono	12	10.0
Standard	86	71.7
TAS	8	6.7
Evaluation		
NCR	4	3.3
CR	40	33.3
PaR	73	60.8
Missing value	3	2.5
Delay to surgery		
Median (min-max)	2 (1–10) mois	
Missing value	58	48.3
Surgery		
Patey	99	82.5
Tumorectomy	20	16.7
Missing value	1	0.8
TPCR		
No	79	65.8
Yes	40	33.3
Missing value	1	0.8
NPCR		
No	58	48.3
Yes	62	51.7
SBR		
I	2	1.7
II	72	60.0
III	25	20.8
Missing value	21	17.5
LVI		
LVI-	45	37.5
LVI+	38	31.7
Missing value	37	30.8
cT stage		
T1C	1	0.8
T2	13	10.8
T3	20	16.7
T4A	2	1.7
T4B	57	47.5
T4C	2	1.7
T4D	20	16.7
Missing value	5	4.2
cN stage		
N0	49	40.8
N1	50	41.7
N2	14	11.7
N3	3	2.5
Missing value	4	3.3
pT stage		
T0	32	26.7
T1	12	10.0
T1A	2	1.7
T2	46	38.3
T3	23	19.2
T4B	2	1.7
Missing value	3	2.5
pN stage		
N0	61	50.8
N1	24	20.0
N2	21	17.5
N3	12	10.0
Missing value	2	1.7
Sataloff T		
TA	36	30.0
TB	44	36.7
TC	28	23.3
TD	12	10.0
Sataloff N		
NA	55	45.8
NB	22	18.3
NC	26	21.7
ND	17	14.2

**Table 2.** Univariate and multivariate logistic regression of BC patients related to general PCR.

Characteristic	Univariate			Multivariable		
	OR	95% CI	*p*-value	OR	95% CI	*p*-value
Sex						
Male	—	—				
Female	8,276,856	0.00, NA	>0.9			
Age	0.98	0.95, 1.02	0.4			
Laterality						
Bilateral	—	—				
Right	0.00		>0.9			
Left	0.00		>0.9			
Histology						
Ductal	—	—				
Lobular	0.92	0.19, 4.87	>0.9			
ER	0.13	0.04, 0.42	<0.001	0.00	0.00, 1.84	0.2
PR	0.14	0.03, 0.64	0.013	2.03	0.00, 127	0.8
HER2						
HER2-	—	—		—	—	
HER2+	2.27	1.01, 5.37	0.052	0.28	0.00, 8.05	0.5
IHC						
HER2+	—	—				
Luminal A	0.10	0.01, 0.61	0.019			
Luminal B	0.34	0.10, 0.98	0.057			
TN	0.87	0.20, 3.76	0.9			
Group						
GB	—	—				
GA	1.86	0.84, 4.20	0.13			
Protocol						
Mixed	—	—				
Mono	1.40	0.30, 6.90	0.7			
Standard	1.66	0.52, 5.26	0.4			
TAS	0.33	0.04, 2.06	0.3			
Evaluation						
NCR	—	—				
CR	6.00	0.69, 128	0.14			
PaR	3.44	0.42, 71.4	0.3			
Delay to surgery	0.68	0.43, 0.97	0.059	0.32	0.01, 1.42	0.3
Surgery						
Patey	—	—				
Tumorectomy	1.45	0.55, 4.16	0.5			
SBR						
I	—	—				
II	0.00		>0.9			
III	0.00		>0.9			
LVI						
Negative	—	—				
Positive	0.66	0.27, 1.56	0.3			
cT stage						
T1C	—	—				
T2	0.00		>0.9			
T3	0.00		>0.9			
T4A	0.00	0.00, 670	>0.9			
T4B	0.00		>0.9			
T4C	0.00		>0.9			
T4D	0.00		>0.9			
cN stage						
N0	—	—				
N1	0.66	0.29, 1.47	0.3			
N2	0.32	0.09, 1.08	0.073			
N3	9,087,887	0.00, NA	>0.9			
pN stage						
N0	—	—		—	—	
N1	0.01	0.00, 0.03	<0.001	0.00		>0.9
N2	0.00	0.00, 0.01	<0.001	0.00		>0.9
N3	0.00	0.00, 0.02	<0.001	0.00		>0.9
Sataloff T						
TA	—	—				
TB	0.00	0.00, 29	>0.9			
TC	0.00	0.00, 2,972	>0.9			
TD	0.00	0.00, 406	>0.9			
Sataloff N						
NA	—	—				
NB	0.00		>0.9			
NC	0.00	0.00, 1,561	>0.9			
ND	0.00	0.00, 449	>0.9			


OR = Odds ratio, CI = Confidence interval

**Table 3.** Univariate and multivariate logistic regression of BC patients related to lymph nodes PCR.

Characteristic	Univariate			Multivariate		
	OR^a^	95% CI^b^	*p*-value	OR^a^	95% CI^b^	*p*-value
Sex						
M	—	—				
F	2,345,128	0.00, NA	>0.9			
Age	0.99	0.96, 1.02	0.4			
Laterality						
Bilateral	—	—				
Right	0.00		>0.9			
Left	0.00		>0.9			
Histology						
Ductal	—	—				
Lobular	1.22	0.26, 6.42	0.8			
ER	0.22	0.07, 0.70	0.011	0.23	0.05, 0.94	0.047
PR	0.23	0.05, 1.00	0.056			
HER2						
HER2-	—	—		—	—	
HER2+	2.35	1.07, 5.36	0.037	0.66	0.18, 2.17	0.5
IHC						
HER2+	—	—				
Luminal A	0.17	0.02, 0.93	0.056			
Luminal B	0.41	0.14, 1.11	0.086			
TN	1.17	0.31, 4.48	0.8			
Group						
GB	—	—				
GA	1.53	0.69, 3.43	0.3			
Protocol						
Mixed	—	—				
Mono	1.40	0.30, 6.90	0.7			
Standard	1.21	0.38, 3.81	0.7			
TAS	0.14	0.01, 1.12	0.10			
Evaluation						
NCR	—	—				
CR	3.67	0.43, 77.7	0.3			
PaR	2.92	0.36, 60.5	0.4			
Delay to surgery	0.76	0.49, 1.07	0.2			
Surgery						
Patey	—	—				
Tumorectomy	1.97	0.74, 5.64	0.2			
SBR						
I	—	—				
II	1.06	0.04, 27.4	>0.9			
III	1.08	0.04, 29.5	>0.9			
LVI						
LVI-	—	—				
LVI+	0.77	0.32, 1.84	0.6			
cT						
T1C	—	—				
T2	0.00		>0.9			
T3	0.00		>0.9			
T4A	0.00	0.00, NA	>0.9			
T4B	0.00		>0.9			
T4C	0.00	0.00, NA	>0.9			
T4D	0.00		>0.9			
cN						
N0	—	—				
N1	0.54	0.24, 1.20	0.13			
N2	0.28	0.07, 0.95	0.051			
N3	10,794,042	0.00, NA	>0.9			
pN						
N0	—	—				
N1	0.00	0.00, 0.01	<0.001			
N2	0.00		>0.9			
N3	0.00		>0.9			
Sataloff T						
TA	—	—		—	—	
TB	0.15	0.05, 0.42	<0.001	0.16	0.03, 0.70	0.021
TC	0.11	0.03, 0.34	<0.001	0.06	0.01, 0.30	0.001
TD	0.07	0.01, 0.29	<0.001	0.04	0.00, 0.26	0.002
Sataloff N						
NA	—	—				
NB	0.00		>0.9			
NC	0.00	0.00, NA	>0.9			
ND	0.00		>0.9			

a: Odds Ratio, b: Confidence Interval

**Table 4.** Univariate and multivariate logistic regression of BC patients related to TPCR.

Characteristic	Univariate			Multivariate		
	OR^a^	95% CI^b^	*p*-value	OR^a^	95% CI^b^	*p*-value
Sex						
F	—	—				
M	0.00		>0.9			
Age	0.97	0.93, 1.00	0.082			
Laterality						
Bilateral	—	—				
Right	0.00		>0.9			
Left	0.00		>0.9			
Histology						
Ductal	—	—				
Lobular	2.74	0.58, 14.5	0.2			
ER	0.08	0.01, 0.33	0.001	0.07	0.00, 4.28	0.2
PR	0.02	0.00, 0.17	0.004	0.07	0.00, 3.54	0.2
HER2						
HER2-	—	—		—	—	
HER2+	4.66	2.06, 10.9	<0.001	1.76	0.12, 27.5	0.7
IHC						
HER2+	—	—		—	—	
Luminal A	0.00		>0.9	0.00		>0.9
Luminal B	0.20	0.07, 0.56	0.003	0.94	0.02, 73.0	>0.9
TN	0.23	0.06, 0.84	0.030	0.10	0.00, 3.64	0.2
Group						
GB	—	—		—	—	
GA	4.06	1.54, 12.8	0.008	5.25	0.66, 112	0.2
Protocol						
Mixed	—	—		—	—	
Mono	2.60	0.22, 60.8	0.5	0.19	0.01, 7.75	0.3
Standard	9.10	1.69, 169	0.037			
TAS	4.33	0.35, 105	0.3	4.38	0.10, 202	0.4
Evaluation						
NCR	—	—				
CR	22,498,831	0.00, NA	>0.9			
PaR	3,713,882	0.00, NA	>0.9			
Delay to surgery	0.66	0.38, 1.03	0.11			
Surgery						
Patey	—	—				
Tumorectomy	0.60	0.18, 1.70	0.4			
SBR						
I	—	—				
II	0.47	0.02, 12.2	0.6			
III	0.47	0.02, 12.9	0.6			
LVI						
LVI-	—	—				
LVI+	0.59	0.22, 1.49	0.3			
Ct						
T1C	—	—				
T2	0.00		>0.9			
T3	0.00		>0.9			
T4A	0.00	0.00, NA	>0.9			
T4B	0.00		>0.9			
T4C	0.00		>0.9			
T4D	0.00		>0.9			
cN						
N0	—	—				
N1	1.00	0.43, 2.31	>0.9			
N2	0.51	0.11, 1.92	0.4			
N3	3.76	0.34, 84.5	0.3			
pN						
N0	—	—				
N1	0.22	0.07, 0.64	0.008			
N2	0.04	0.00, 0.22	0.003			
N3	0.08	0.00, 0.48	0.022			
Sataloff T						
TA	—	—				
TB	0.00	0.00, NA	>0.9			
TC	0.00	0.00, NA	>0.9			
TD	0.00		>0.9			
Sataloff N						
NA	—	—		—	—	
NB	0.11	0.02, 0.35	<0.001	0.03	0.00, 0.28	0.010
NC	0.09	0.02, 0.30	<0.001	0.04	0.00, 0.33	0.010
ND	0.04	0.00, 0.23	0.003	0.02	0.00, 0.27	0.011

a: Odds Ratio, b: Confidence Interval
